# Unraveling the nexus of age, epilepsy, and mitochondria: exploring the dynamics of cellular energy and excitability

**DOI:** 10.3389/fphar.2024.1469053

**Published:** 2024-09-05

**Authors:** Wen Xie, Sushruta Koppula, Mayur B. Kale, Lashin S. Ali, Nitu L. Wankhede, Mohit D. Umare, Aman B. Upaganlawar, Ahmed Abdeen, Elturabi E. Ebrahim, Mohamed El-Sherbiny, Tapan Behl, Bairong Shen, Rajeev K. Singla

**Affiliations:** ^1^ Department of Pharmacy and Institutes for Systems Genetics, Center for High Altitude Medicine, Frontiers Science Center for Disease-related Molecular Network, West China Hospital, Sichuan University, Chengdu, China; ^2^ College of Biomedical and Health Sciences, Konkuk University, Chungju-Si, Republic of Korea; ^3^ Smt. Kishoritai Bhoyar College of Pharmacy, Kamptee, India; ^4^ Department of Basic Medical Sciences, Faculty of Dentistry, Al-Ahliyya Amman University, Amman, Jordan; ^5^ SNJB’s Shriman Sureshdada Jain College of Pharmacy, Nasik, India; ^6^ Department of Forensic Medicine and Toxicology, Faculty of Veterinary Medicine, Benha University, Toukh, Egypt; ^7^ Medical-Surgical Nursing Department, College of Applied Medical Sciences, Prince Sattam bin Abdulaziz University, Al-Kharj, Saudi Arabia; ^8^ Department of Basic Medical Sciences, College of Medicine, AlMaarefa University, Riyadh, Saudi Arabia; ^9^ Department of Anatomy, Faculty of Medicine, Mansoura University, Mansoura, Egypt; ^10^ Amity School of Pharmaceutical Sciences, Amity University, Mohali, India; ^11^ Institutes for Systems Genetics, West China Tianfu Hospital, Frontiers Science Center for Disease-related Molecular Network, West China Hospital, Sichuan University, Chengdu, China; ^12^ School of Pharmaceutical Sciences, Lovely Professional University, Phagwara, India

**Keywords:** epilepsy, ageing, epigenetic modification, ketogenic diet, mitochondria targeted therapy

## Abstract

Epilepsy, a complex neurological condition marked by recurring seizures, is increasingly recognized for its intricate relationship with mitochondria, the cellular powerhouses responsible for energy production and calcium regulation. This review offers an in-depth examination of the interplay between epilepsy, mitochondrial function, and aging. Many factors might account for the correlation between epilepsy and aging. Mitochondria, integral to cellular energy dynamics and neuronal excitability, perform a critical role in the pathophysiology of epilepsy. The mechanisms linking epilepsy and mitochondria are multifaceted, involving mitochondrial dysfunction, reactive oxygen species (ROS), and mitochondrial dynamics. Mitochondrial dysfunction can trigger seizures by compromising ATP production, increasing glutamate release, and altering ion channel function. ROS, natural byproducts of mitochondrial respiration, contribute to oxidative stress and neuroinflammation, critical factors in epileptogenesis. Mitochondrial dynamics govern fusion and fission processes, influence seizure threshold and calcium buffering, and impact seizure propagation. Energy demands during seizures highlight the critical role of mitochondrial ATP generation in maintaining neuronal membrane potential. Mitochondrial calcium handling dynamically modulates neuronal excitability, affecting synaptic transmission and action potential generation. Dysregulated mitochondrial calcium handling is a hallmark of epilepsy, contributing to excitotoxicity. Epigenetic modifications in epilepsy influence mitochondrial function through histone modifications, DNA methylation, and non-coding RNA expression. Potential therapeutic avenues targeting mitochondria in epilepsy include mitochondria-targeted antioxidants, ketogenic diets, and metabolic therapies. The review concludes by outlining future directions in epilepsy research, emphasizing integrative approaches, advancements in mitochondrial research, and ethical considerations. Mitochondria emerge as central players in the complex narrative of epilepsy, offering profound insights and therapeutic potential for this challenging neurological disorder.

## Graphical Abstract

**Figure:**
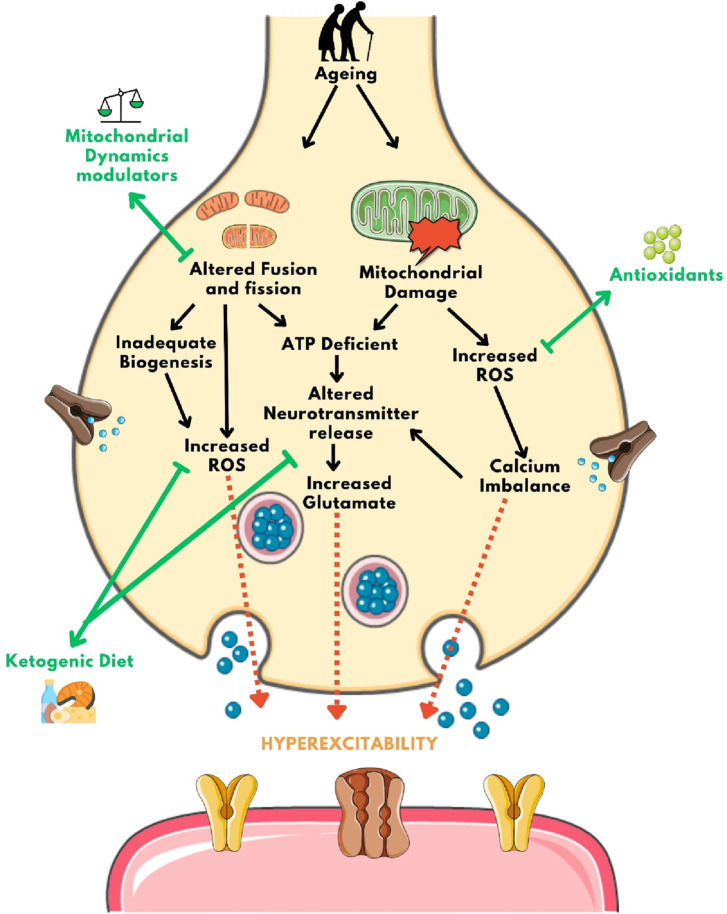

## 1 Introduction

Epilepsy affects millions globally and is characterized by recurrent, unprovoked seizures. These seizures stem from abnormal electrical activity in the brain and can present in various forms, ranging from brief lapses in consciousness to full-body convulsions (Abramovici and Bagić, 2016; Chen et al., 2022). Epilepsy has far-reaching implications, including cognitive and emotional disturbances, social stigmatization, and a substantial reduction in the quality of life for those affected (Vrinda et al., 2019). The etiology of epilepsy is diverse, with both genetic and acquired factors contributing to its development. Despite the diversity in causative factors, many cases share standard features at the cellular and molecular levels, making it an exciting area of research (Falco-Walter, 2020). When compared to young people and older people, epilepsy affects the elderly more frequently and is a somewhat prevalent condition in this demographic (Lee, 2019). Epilepsy tends to occur with age, not only because aging itself might be a trigger for seizures but also because several epileptogenic disorders are age-related. Considering such factors, an increase in the prevalence of epilepsy can be linked to the continuous growth and aging of the global population throughout time (Vrinda et al., 2019; Piccenna et al., 2023; Brodie and Kwan, 2005).

Mitochondria, commonly known as the “powerhouses of the cell,” are crucial for cellular energy regulation. These double-membraned organelles generate adenosine triphosphate (ATP), the primary energy currency of cells. By undergoing a series of enzymatic reactions within the mitochondrial matrix, energy-rich molecules like glucose and fatty acids are metabolized to produce ATP through oxidative phosphorylation (Angelova and Abramov, 2018). ATP is essential for many cellular processes, including muscle contraction, ion channel regulation, and neurotransmitter release. Neurons, in particular, have a high demand for energy due to their constant electrical activity and neurotransmission. Thus, maintaining mitochondrial function is critical for regular brain activity (Kann and Kovács, 2007). Mitochondria are now understood to be more than just primary bioenergetic factories; instead, they are sites for signaling molecules, critical components of the natural immune system, and stem cell activity regulators. All of these features, furthermore, offer insights into how mitochondria may control aging and diseases associated with age (Sun et al., 2016).

The intriguing connection between mitochondria and neuronal excitability has emerged as a focal point in epilepsy research. Neuronal excitability refers to the propensity of neurons to generate electrical impulses, a fundamental aspect of brain function (Alberti et al., 2022). This excitability is tightly regulated to ensure the delicate equilibrium of inhibition and excitation in the brain, maintaining stable neural networks (Kann and Kovács, 2007). Mitochondrial dysfunction can profoundly impact neuronal excitability. When mitochondria fail to produce sufficient ATP or improperly regulate calcium levels, neurons become vulnerable to hyperexcitability. This hyperexcitability can manifest as increased spontaneous firing of action potentials, making it easier for seizures to occur (Missiroli et al., 2020). ATP binding to KATP channels keeps them closed, preventing excessive neuronal firing (Xiao et al., 2023). However, when ATP levels are low due to mitochondrial dysfunction, these channels open, leading to hyperexcitability and potentially seizure activity (Huang and Afawi, 2011). Additionally, mitochondria are intimately involved in calcium regulation within neurons (Matuz-Mares et al., 2022). Elevated intracellular calcium levels can trigger a cascade of events, including releasing neurotransmitters and activating signaling pathways that contribute to hyperexcitability. Mitochondria help buffer and regulate calcium levels, and when they malfunction, this regulation is disrupted, further exacerbating excitability (Loewen et al., 2016).

## 2 Mitochondrial function in neurons

Mitochondria emerge as central players in the intricate tapestry of neurological function, orchestrating a symphony of events critical for neuronal health and vitality (Aguiar et al., 2012). The neuronal mitochondrial population is not static; it undergoes fission and fusion processes to adapt to changing energy needs and maintain mitochondrial health. Mitochondrial physiology is characterized by a series of biochemical reactions within their matrix. The Krebs cycle and oxidative phosphorylation are central to their function. During these processes, mitochondria metabolize substrates, such as glucose and fatty acids, to produce ATP, the primary energy source for neurons (Eckert and Pagani, 2011; Badole et al., 2021; Kale et al., 2020).

It is not unexpected that disorders that vary from minor changes in the activity of neurons to cell death and neurodegeneration are caused by disruptions in the brain’s energy metabolisms (Rink and Khanna, 2011). The research of age-associated mitochondrial deficits is gaining attention to understand the process contributing towards either normal aging or neurodegenerative illnesses, considering the pivotal role mitochondria play regarding energy consumption and controlling redox equilibrium (Brand et al., 2013).

### 2.1 ATP production and energy metabolism in neurons

Mitochondria fulfill this energy demand by generating ATP through oxidative phosphorylation. This process entails the transfer of electrons across several protein complexes within the inner membrane of mitochondria, ultimately leading to ATP synthesis (Xavier et al., 2016; Umare et al., 2021). Notably, neurons exhibit diverse energy demands based on their activity levels and location within the brain. Synaptic terminals, for instance, require rapid ATP production to support neurotransmission. Mitochondria are strategically positioned at synapses to meet this need promptly. Moreover, during increased neuronal activity, mitochondria move along axons and dendrites to the sites of higher energy consumption, ensuring a continuous energy supply (Tiwari et al., 2021; Brown et al., 2006).

### 2.2 Calcium homeostasis and mitochondria

Calcium ions (Ca^2+^) are pivotal in neuronal signaling and neurotransmitter release. Maintaining precise control over intracellular calcium concentrations is essential to prevent excessive excitability and excitotoxicity (Beal, 1995; Gibson et al., 2010). When neurons experience increased calcium influx, as occurs during neurotransmission, mitochondria buffer excess calcium ions, preventing their accumulation in the cytosol (Rharass et al., 2014; Panov et al., 2002). This buffering action helps dampen excitatory signals and leads to synaptic event termination (D’Angelo et al., 2023). Moreover, mitochondria sequester calcium within their matrix, where it can be safely stored and released as needed. This calcium-handling capability of mitochondria is particularly relevant in the context of epilepsy. Dysfunctional mitochondria can lead to disrupted calcium regulation, potentially resulting in heightened neuronal excitability and an increased susceptibility to seizures (Matuz-Mares et al., 2022). Wide harmful events, including elevated ROS generation and abnormalities in the control of intracellular calcium concentrations, have been linked to a critical role for mitochondria in aging-related diseases. These events have been linked to mPTP activation, which has significant implications for cell viability. Consequently, mPTP becomes a viable strategy for neuroprotection in neurodegenerative disorders associated with aging (Baev et al., 2022; Pivovarova and Andrews, 2010; Baev et al., 2024).

Cells can adjust and operate in a constantly evolving cellular setting by establishing interaction among organelles. At particular locations known as mitochondria-associated membranes (MAMs), ER and mitochondria combine to regulate several cellular processes, like lipid generation and transfer, apoptosis, mitochondrial dynamics, and calcium signaling (Saneto and Perez, 2022). Furthermore, these activities are notably impacted early in the pathophysiology of neurodegenerative conditions, indicating a potential role for MAMs in the etiology of these conditions (Kim et al., 2022).

## 3 Mitochondrial Dysfunction in Epilepsy

Mitochondrial dysfunction in epilepsy represents a critical nexus where cellular energy regulation and excitability intersect. This section delves into the intricate relationship between epilepsy and mitochondrial dysfunction, with a focus on its role in seizure generation, the impact of ROS, and the influence of genetic and environmental factors on mitochondrial function in epilepsy (Kann and Kovács, 2007; Aguiar et al., 2012; Shin et al., 2011; Upaganlawar et al., 2021).

**FIGURE 1:**
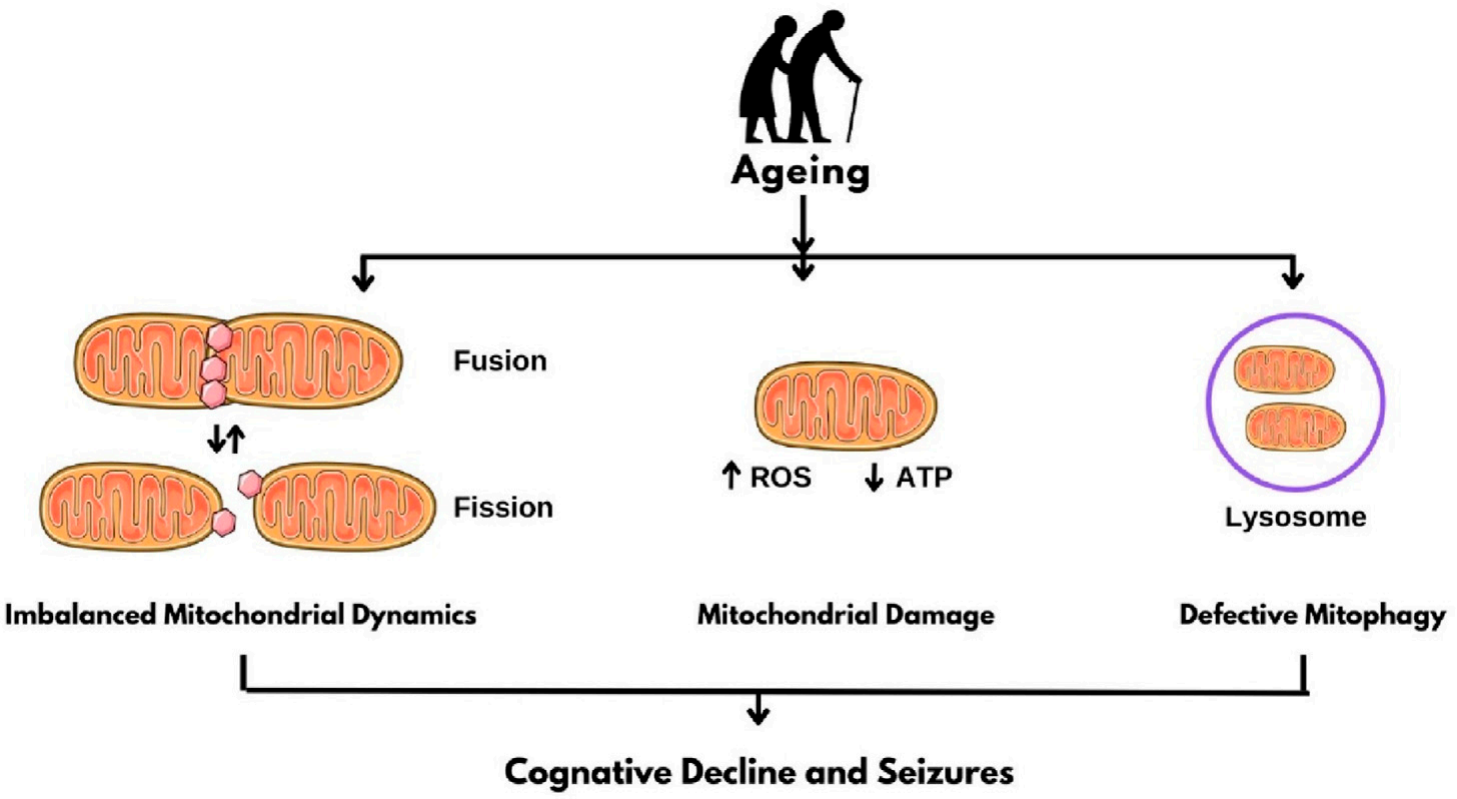
Mitochondrial Dysfunction in Epilepsy. ROS: Reactive oxygen species; ATP: Adenosine triphosphate.

As individuals age, they encounter a growing number of risk factors for seizures and epilepsy due to the higher prevalence of comorbidities compared to children and adults. Various age-related diseases are linked to seizures, including Alzheimer’s disease and other dementias, stroke, and other vascular conditions, as well as several metabolic disorders, primarily diabetes and electrolyte imbalances (Figure 1) (Liu et al., 2016)

### 3.1 Role of mitochondrial dysfunction in seizure generation

Mitochondrial dysfunction has emerged as a compelling contributor to the genesis of seizures in epilepsy. This subsection delves into the intricate mechanisms through which impaired mitochondrial function can fuel hyperexcitability and provide a fertile ground for seizure initiation (Shin et al., 2011).

#### 3.1.1 ATP depletion

Neurons have an insatiable appetite for energy due to their continuous electrical activity, neurotransmitter synthesis and release, and the maintenance of ion gradients across their membranes (Chang and Reynolds, 2006). When mitochondrial function is compromised, as can occur in various forms of mitochondrial dysfunction, ATP production is hampered. This reduction in ATP levels can have profound consequences for neuronal excitability (V Terry et al., 2023). Neurons rely on ATP-dependent pumps and channels to maintain ion gradients necessary for normal membrane potential. When ATP becomes scarce, these pumps and channels struggle to function optimally (Pivovarov et al., 2019).This situation leads to the depolarization of the neuronal membrane, reducing the threshold for spontaneous action potential firing. Essentially, ATP depletion due to mitochondrial dysfunction renders neurons more excitable, increasing their susceptibility to spontaneous, uncontrolled electrical discharges–the hallmark of seizures (Pearson-Smith and Patel, 2017; Waldbaum and Patel, 2010a).

#### 3.1.2 Enhanced glutamate release

Another facet of mitochondrial dysfunction’s impact on seizure generation is its effects on calcium homeostasis within neurons. Mitochondria are crucial for buffering and regulating calcium levels within cells. However, when mitochondrial function falters, this regulation becomes compromised. Elevated intracellular calcium concentrations can lead to excessive glutamate release at excitatory synapses (Dong et al., 2009). Glutamate is the primary excitatory neurotransmitter in the CNS, and its release activates postsynaptic receptors, leading to neuronal excitation (Lewerenz and Maher, 2015; Kim et al., 2020; Aglawe et al., 2021). When mitochondria fail to buffer and control calcium levels efficiently, it can trigger an aberrant release of glutamate, leading to synaptic overactivity. This glutamate excess can propagate as a wave of excitation, potentially culminating in the synchronized firing of a population of neurons–a seizure. (Verma et al., 2022).

#### 3.1.3 Altered ion channel function

Mitochondrial dysfunction can also perturb the function of ion channels in neurons, further exacerbating hyperexcitability. One critical set of ion channels affected by this dysfunction is the ATP-sensitive potassium (KATP) channels. KATP channels play a vital role in regulating neuronal excitability (Nikbakht et al., 2021).Typically, ATP inhibits them, and their closure leads to membrane hyperpolarization and reduced excitability (O’Rourke, 2000). However, in mitochondrial dysfunction, where ATP levels are diminished, these channels may fail to remain closed. This failure allows potassium ions to flow out of the neuron, leading to membrane depolarization and increased neuronal excitability (Walz, 2003). In essence, mitochondrial dysfunction can indirectly affect the behavior of ion channels, like KATP channels, exacerbating hyperexcitability and potentially facilitating the onset of seizures (Picca et al., 2017).

### 3.2 Impact of ROS in epileptogenesis

ROS are decidedly reactive molecules that contain free radicals like superoxide (O_2_^−^) and hydrogen peroxide (H_2_O_2_). They are natural byproducts of various cellular processes, including mitochondrial respiration. While ROS plays vital roles in cell signaling and immune defense under normal circumstances, an excessive build-up of ROS, often associated with mitochondrial dysfunction, can have profound implications for epileptogenesis, leading to epilepsy (Upaganlawar et al., 2021; Waldbaum and Patel, 2010a; Dong et al., 2009).

#### 3.2.1 Oxidative stress

Mitochondrial dysfunction, frequently observed in epilepsy, can upset the balance between ROS production and the body’s antioxidant defenses. This imbalance results in oxidative stress, a condition where ROS levels exceed the cell’s ability to neutralize them. Oxidative stress can have harmful effects on neurons (Umare et al., 2021; Upaganlawar et al., 2021). Oxidative stress can harm lipids, proteins, and DNA, leading to neuronal dysfunction and potentially cell death. This damage may induce structural and functional changes in neurons, fostering conditions favorable to epileptogenesis. Additionally, oxidative stress is strongly linked to neuroinflammation, forming a reciprocal relationship. These processes create a feedback loop, each intensifying the other and fostering a pro-epileptogenic setting in the brain (Pearson-Smith and Patel, 2017; Morimoto et al., 2018; Nigar et al., 2016; de Araújo Filho et al., 2018).

All aerobic cells create reactive oxygen and nitrogen species (RONS), which are crucial in aging and age-related illnesses. The oxidative stress hypothesis about aging, formerly known as the free radical theory of aging, is predicated on the structural breakdown theory, which holds that the build-up of oxidative damage to macromolecules causes age-related functional deficits. Movement and cognitive dysfunction impact older people’s longevity and standard of life (Tiwari et al., 2021; Beal, 1995; Liguori et al., 2018; V Mangrulkar et al., 2023).

Several neuropsychiatric disorders, including epilepsy, which can lead to progressive movement impairment and cognitive deterioration or eventual immobility, significantly affect older individuals. Research has shown that cellular oxidative damage plays a role in the development of dementia and seizures. Numerous studies have investigated the relationship between cognitive function, as assessed by the Mini-Mental State Examination (MMSE), and levels of various indicators of ROS (Mrakic-Sposta et al., 2018).

#### 3.2.2 Neuronal excitability

ROS can influence the function of ion channels and receptors that regulate neuronal excitability. One important target is the NMDA receptor, a glutamate receptor subtype essential for synaptic plasticity and excitatory neurotransmission (Dong et al., 2009; Verma et al., 2022; Vezzani et al., 2016). ROS can enhance the activity of NMDA receptors, making them more responsive to glutamate. Heightened NMDA receptor activity can increase calcium influx into neurons (Liu et al., 2013; Sluka et al., 1985; John et al., 2022). Elevated intracellular calcium concentrations can lead to excitotoxicity. Furthermore, ROS can influence other ion channels and receptors that affect neuronal membrane potential and excitability. The net effect is often an increased propensity for neurons to fire spontaneously, which can lower the threshold for seizures (de Vrese et al., 2011; Yap and Lye, 2020).

#### 3.2.3 Neuroinflammation

ROS contributes to the initiation and persistence of neuroinflammation, activating immune cells and releasing pro-inflammatory molecules in the brain. Neuroinflammation is increasingly acknowledged as a critical factor in the development of epilepsy (Upaganlawar et al., 2021; Xanthos and Sandkühler, 2014). ROS can activate pro-inflammatory pathways within glial cells (Rauf et al., 2024). Activated cells release cytokines, chemokines, and other inflammatory mediators that can influence neuronal function and connectivity (Xanthos and Sandkühler, 2014; Lyman et al., 2014; Kovács et al., 2014). Additionally, neuroinflammation can result in BBB dysfunction, allowing immune cells from the bloodstream to infiltrate the brain. This immune cell infiltration and the ensuing inflammatory response can further contribute to neuronal hyperexcitability and promote epileptogenesis (Okuneva et al., 2016; Beaurain et al., 2019).

## 4 Mitochondrial dynamics in epileptic brain

### 4.1 Mitochondrial fusion and fission: Cellular implications

Mitochondrial dynamics involve two opposing processes: fusion and fission (Picca et al., 2021; Lee and Yoon, 2016). Mitochondrial fusion merges individual mitochondria into a single, interconnected network. This mechanism facilitates the transfer of mitochondrial contents, such as proteins and DNA, promoting the mingling of healthy and impaired mitochondria. Fusion additionally supports the preservation of an optimal mitochondrial membrane potential and the restoration of damaged mitochondrial DNA. It ensures a uniform mitochondrial population, vital for effective energy generation and calcium regulation (Chakravorty et al., 2019; Suen et al., 2008; Wankhede et al., 2022). Mitochondrial Fission, conversely, partitions mitochondria into smaller organelles. This mechanism is vital for quality control, enabling the segregation and elimination of impaired or dysfunctional mitochondria. Fission also aids in distributing mitochondria to regions with elevated energy requirements, such as synaptic terminals (Chen et al., 2023). Collectively, fusion and fission uphold a dynamic balance of mitochondrial structure and function, pivotal for cellular homeostasis (Badole et al., 2021; Suárez-Rivero et al., 2017).

### 4.2 Altered mitochondrial dynamics in epilepsy

**FIGURE 2:**
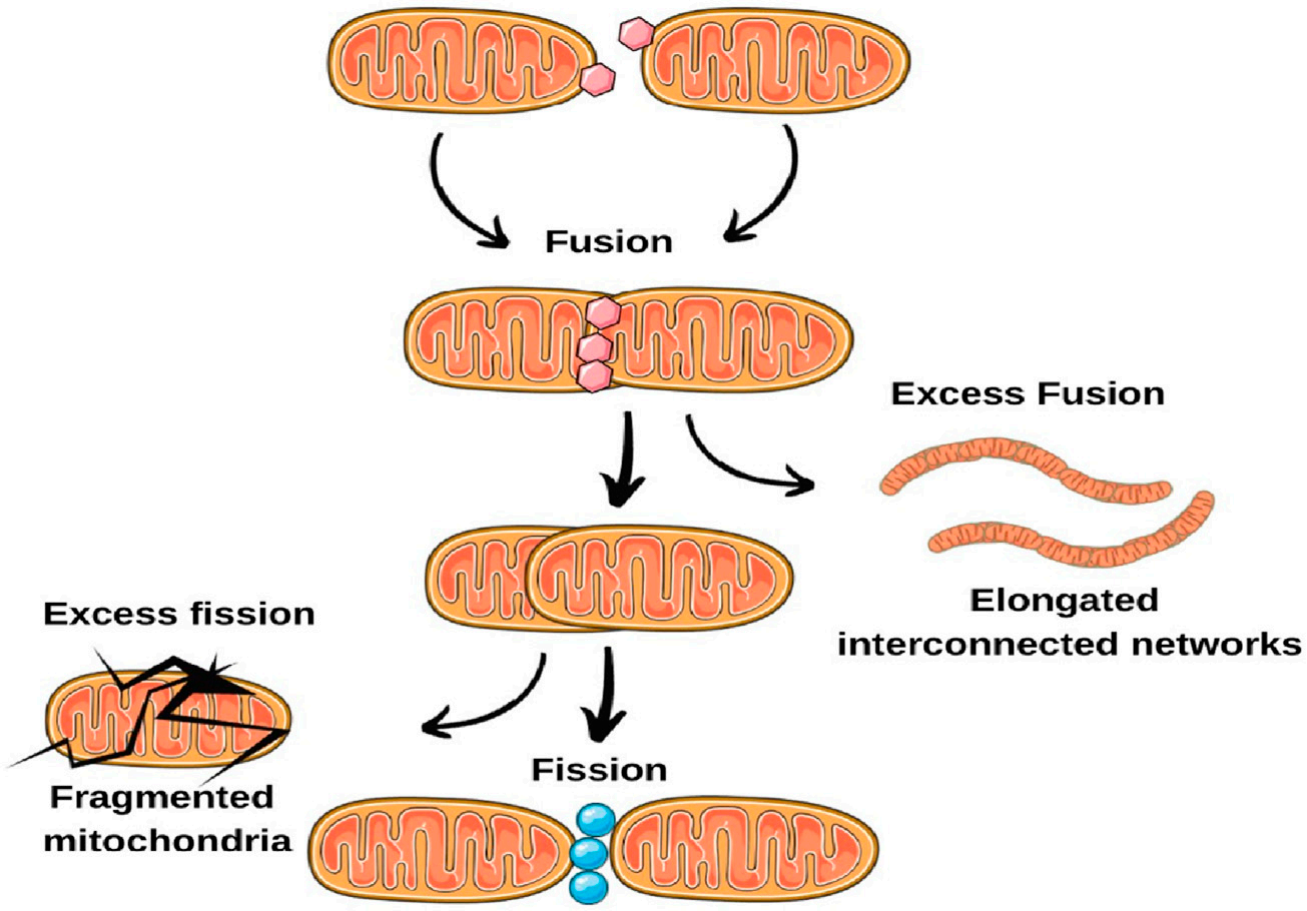
Imbalances in mitochondrial fusion and fission dynamics.

Recent findings indicate disruptions in mitochondrial dynamics in epilepsy. These disruptions can carry substantial implications for neuronal wellbeing and excitability. Investigations using animal models of epilepsy and *postmortem* brain tissue from epilepsy patients have unveiled discrepancies in the regulation of mitochondrial fusion and fission dynamics (Olkhova et al., 2023). In certain instances, an abundance of fission occurs, resulting in fragmented mitochondria, whereas in other cases, fusion prevails, yielding elongated, interconnected networks. These imbalances can interfere with mitochondrial quality control, impede the elimination of impaired mitochondria, and undermine overall mitochondrial function (Kundap et al., 2020). Further, proper mitochondrial trafficking along axons and dendrites is essential for meeting the energy demands of specific neuronal regions (Chang and Reynolds, 2006; Gao et al., 2017; Schwarz, 2013). Changes in fusion-fission dynamics can disturb mitochondrial trafficking, leading to irregular distribution and compromised energy supply to vital regions. This disparity in mitochondrial distribution might contribute to heightened neuronal hyperexcitability, a characteristic feature of epilepsy (Reddy et al., 2011; Bossy-Wetzel et al., 2008; Chen and Chan, 2009). Altered mitochondrial dynamics can also affect calcium regulation within neurons. Mitochondria participate in buffering intracellular calcium levels, and disruptions in fusion and fission events can lead to imbalances in calcium handling. Elevated intracellular calcium concentrations can enhance neuronal excitability, making neurons more susceptible to seizures (Figure 2).

### 4.3 Role of mitochondrial dynamics in seizure propagation

The interaction between mitochondrial dynamics and epilepsy represents a multifaceted area of investigation, drawing increasing attention within the field of epilepsy research. Neurons, known for their voracious energy demands, depend heavily on the finely tuned orchestration of mitochondrial dynamics to maintain essential cellular processes. In the context of epilepsy, these dynamics play a pivotal yet intricate role in seizure propagation (Suárez-Rivero et al., 2017). Age-related disorders and aging have been consistently linked to aberrant mitochondrial architecture, suggesting that cellular dynamics are degraded with cellular aging (Bhatti et al., 2017). Several investigations indicate that mitochondrial activity effectively regulates the aging process in addition to pathological conditions. Moreover, alterations to the structure or function of mitochondria can directly impact an organism’s lifetime (Guo et al., 2022).

#### 4.3.1 Mitochondrial health and seizure threshold

Central to the discussion is that well-functioning mitochondria are paramount for neurons to maintain normal membrane potential and energy equilibrium. Perturbations in mitochondrial dynamics, resulting in mitochondrial fragmentation or dysfunction, have been observed in animal models and human epilepsy cases (Lee et al., 2004; Walker et al., 2020). In such instances, neurons are more susceptible to hyperexcitability. Heightened neuronal excitability effectively lowers the threshold for seizure initiation and propagation. Studies have highlighted the importance of mitochondrial fusion and fission events in modulating neuronal excitability. Imbalances in these processes can disrupt the normal functioning of mitochondria, contributing to altered membrane potential dynamics. Consequently, neurons with compromised mitochondria are more prone to firing spontaneously, a hallmark of epileptic seizures (Flippo and Strack, 2017; Marde et al., 2022; Marde et al., 2021).

#### 4.3.2 Calcium buffering and excitotoxicity

**FIGURE 3:**
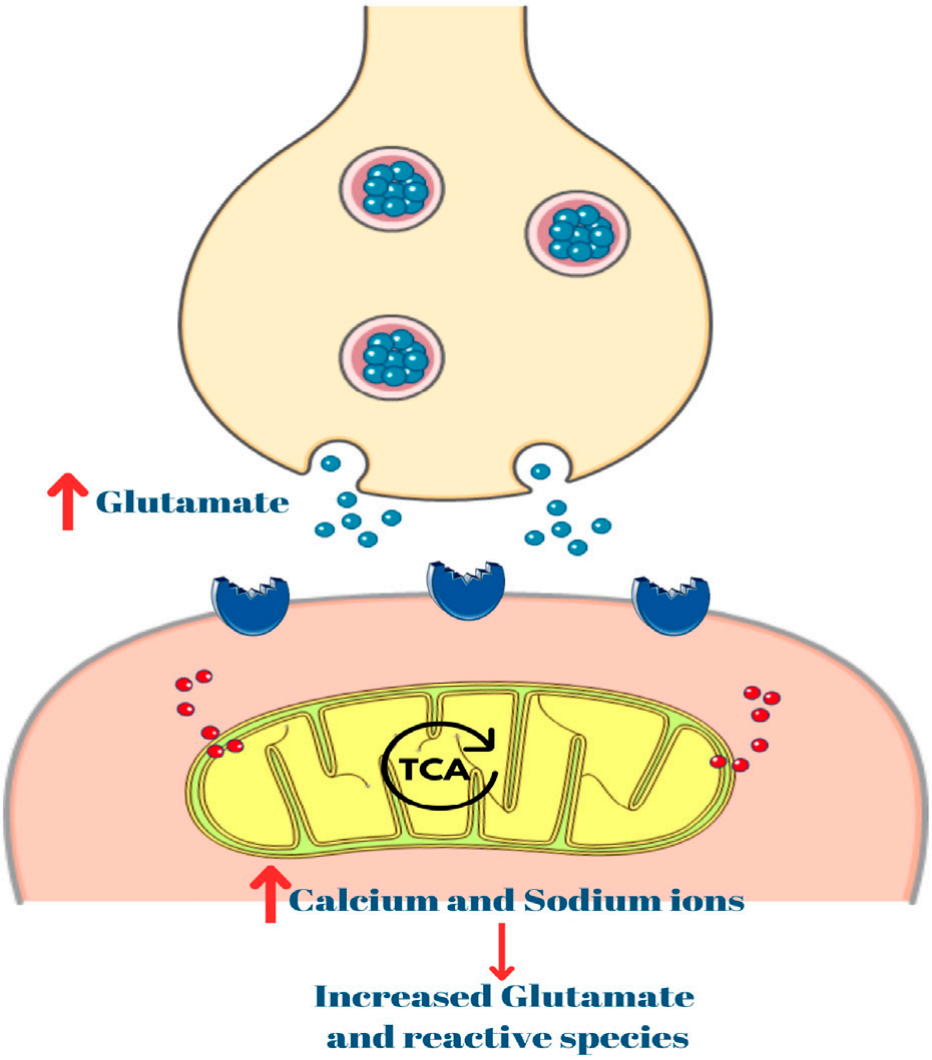
Role of calcium in excitotoxicity. TCA: Tricarboxylic acid.

Mitochondria regulates intracellular calcium levels, a factor intricately linked to neuronal excitability. Dysfunctional mitochondrial dynamics can impair their capacity to buffer calcium ions effectively, leading to unchecked calcium accumulation within neurons during seizures (Zong et al., 2024). Excessive intracellular calcium concentrations, a consequence of impaired calcium buffering, amplify excitotoxicity–a process where overstimulation of glutamate receptors leads to neuronal damage. Heightened excitotoxicity not only contributes to the severity of seizures but also facilitates their spread to neighboring regions (Figure 3) (Rharass et al., 2014; Pivovarova and Andrews, 2010; Valko et al., 2005)

#### 4.3.3 Mitochondrial quality control and seizure-induced stress

During seizures, neurons endure significant metabolic stress due to heightened energy demands. Mitochondrial dynamics become crucial in maintaining mitochondrial quality control under such conditions. Dysfunctional dynamics can hinder the removal of damaged mitochondria from the cell (Xanthos and Sandkühler, 2014; Rho and Boison, 2022). The accumulation of dysfunctional mitochondria intensifies cellular stress and exacerbates the persistence of seizures (Rodolfo et al., 2018; Pickrell et al., 2009). Research from experimental models and genetic studies highlights the significance of mitochondrial dynamics in epilepsy. In experimental epilepsy models, scientists have noted mitochondrial fragmentation and dysfunction in neurons during seizures. Furthermore, changes in genes related to mitochondrial dynamics have been associated with epilepsy (Kalra, 2023; Lehtinen et al., 2009). This convergence of evidence underscores the pivotal role mitochondrial dynamics play in epileptic processes. Higher amounts of oxidized protein molecules, lipids in the membrane, and damaged DNA are factors at the cellular level that cause functional and structural abnormalities, eventually resulting in cell death. Proteotoxic stress and the build-up of oxidized lipids are noteworthy correlations with age-related deficiencies in autophagy (Su et al., 2019).

## 5 Bioenergetics and epileptic seizures

One critical aspect of seizure generation and propagation is the intricate interplay between energy metabolism and neuronal excitability. This section explores the bioenergetics of epileptic seizures, including the energy demands during seizures and the contributions of mitochondria to seizure energetics.

### 5.1 Energy demands during seizures

Despite its relatively small size, the brain is a remarkably energy-intensive organ, consuming a disproportionate amount of the body’s total energy. Neurons, in particular, are voracious energy consumers due to their constant electrical activity, neurotransmitter synthesis and release, and the maintenance of ion gradients across their membranes. Epileptic seizures represent a state of heightened neuronal activity characterized by repetitive and synchronized firing of neurons. This heightened activity places substantial metabolic demands on the brain. The energy demands during seizures are primarily met through the ATP generated by the mitochondria (Vergara et al., 2019).

During seizures, neurons experience a surge in energy demands, primarily to support the multiple processes. Neurons rely on energy-consuming ion pumps, such as the sodium-potassium pump (Na+/K+ pump), to maintain ion gradients across their membranes. These ion gradients are essential for normal neuronal excitability and neurotransmission (Zsurka and Kunz, 2015). During seizures, the Na+/K+ pump works overtime to restore membrane potential, consuming considerable ATP. Additionally, the repetitive firing of action potentials during seizures demands substantial energy. Each action potential requires the active transport of ions across the neuronal membrane, which is energetically costly. Further, releasing neurotransmitters, such as glutamate and GABA, is energy-intensive (Kann and Kovács, 2007; Zsurka and Kunz, 2015; Taksande et al., 2017). These neurotransmitters are packaged into synaptic vesicles and released into the synaptic cleft during neuronal communication. The postsynaptic response to neurotransmitter release involves the activation of ion channels, such as NMDA receptors, which are critical for synaptic plasticity and excitatory neurotransmission. These receptor activations are energy-dependent. Epileptic seizures dramatically increase the energy demands of neurons due to the heightened electrical activity and neurotransmission (Sumadewi et al., 2023). These demands are met primarily through mitochondrial ATP production (Badole et al., 2021; Tiwari et al., 2021; Upaganlawar et al., 2021; Kim et al., 2020).

### 5.2 Mitochondrial contribution to seizure energetics

Mitochondria play a pivotal role in meeting the heightened energy demands of seizures by serving as the primary ATP generators within neurons. Mitochondria exhibit flexibility in utilizing substrates for ATP production. During seizures, the brain resorts to anaerobic glycolysis as an alternative energy source, producing lactate. Subsequently, mitochondria can convert lactate back into pyruvate and employ it for oxidative phosphorylation. This lactate-pyruvate shuttle is essential for sustaining energy production during prolonged seizures (Zsurka and Kunz, 2015). It plays a pivotal role in buffering intracellular calcium levels during seizures. The excessive calcium influx into neurons can lead to excitotoxicity. Mitochondria, with their ability to sequester calcium within their matrix, help prevent this calcium-induced neuronal damage. This calcium buffering is energy-dependent and relies on the electrochemical gradient maintained by mitochondria (Kovac et al., 2017). Mitochondria also contribute to redox balance, which is essential during seizures. The production of ROS is elevated during heightened neuronal activity. Mitochondria are both sources and targets of ROS. They can produce ROS as natural byproducts of respiration, but they also have antioxidant defenses to mitigate oxidative stress. Maintaining this delicate balance is crucial for cellular health during seizures (Umare et al., 2021).

## 6 Excitability and ion channels

Epileptic seizures are characterized by abnormal and synchronized neuronal firing, highlighting the pivotal role of ion channels in regulating neuronal excitability. This section delves into the intricate relationship between excitability, ion channels, and mitochondria in epilepsy.

### 6.1 Ion channels and their regulation in neurons

Neurons have an impressive array of ion channels that meticulously regulate their excitability. These channels fall into several categories, each with unique properties and functions.

#### 6.1.1 Voltage-gated ion channels

These channels, including sodium (Na+), potassium (K^+^), and calcium (Ca^2+^) channels, play a fundamental role in generating action potentials, the electrical signals that transmit information within neurons. Voltage-gated sodium channels initiate and propagate action potentials, while voltage-gated potassium channels are critical for repolarizing and terminating these signals. Voltage-gated calcium channels, particularly the L-type, regulate calcium influx, impacting neurotransmitter release and synaptic plasticity (V Frolov et al., 2016; Lerche et al., 2013).

#### 6.1.2 Ligand-gated ion channels

These channels, such as N-methyl-D-aspartate (NMDA) and gamma-aminobutyric acid (GABA) receptors, are activated by neurotransmitters. NMDA receptors are essential for synaptic plasticity and excitatory neurotransmission, while GABA receptors mediate inhibitory neurotransmission, dampening neuronal excitability (Lerche et al., 2013).

#### 6.1.3 Transient receptor potential (TRP) channels

TRP channels participate in diverse cellular processes, including thermosensation, osmosensation, and nociception. Temperature changes activate some TRP channels, while others respond to various chemical and physical stimuli, contributing to neuronal excitability and sensory perception (Lerche et al., 2013).

#### 6.1.4 Calcium-activated ion channels

Calcium-activated potassium (KCa) channels and calcium-activated chloride channels are channels modulated by intracellular calcium concentrations. They play critical roles in shaping action potentials, regulating synaptic transmission, and modulating neuronal excitability (Lerche et al., 2013).

The activity of ion channels in neurons is tightly regulated to ensure precise control over neuronal excitability. Several mechanisms modulate ion channel function. Protein kinases, such as protein kinase A (PKA) and protein kinase C (PKC), phosphorylate ion channels, altering their conductance and gating properties. This regulation is essential for synaptic plasticity and the fine-tuning of neuronal excitability (Lerche et al., 2013; Chambers and Kramer, 2008). Also, the binding of neurotransmitters to receptors can either enhance or inhibit ion channel activity. For example, glutamate binding to NMDA receptors permits calcium influx, while GABA binding to GABA receptors enhances chloride influx, inhibiting neuronal firing. Furthermore, intracellular signaling pathways modulate ion channel activity, including the cyclic AMP (cAMP) and phosphoinositide pathways. These pathways can be activated by various extracellular signals, further fine-tuning neuronal excitability (Smart and Paoletti, 2012).

### 6.2 Interplay between mitochondria and ion channels in epilepsy

Mitochondria exert a multifaceted influence on neuronal excitability through their intricate interactions with ion channels. Firstly, they serve as the primary energy suppliers, generating ATP through oxidative phosphorylation, which is essential for operating ion pumps like the sodium-potassium (Na^+^/K^+^) pump (Baev et al., 2022). Reduced mitochondrial ATP production can disrupt ion balance, leading to neuronal hyperexcitability (Clemente-Suárez et al., 2023). Secondly, mitochondria act as vital regulators of intracellular calcium levels, efficiently buffering elevated calcium concentrations that can activate calcium-sensitive ion channels, including calcium-activated potassium (KCa) channels. This calcium buffering function helps maintain proper ion channel activity and prevents aberrant neuronal excitability (Kann and Kovács, 2007; Kovac et al., 2017; V Frolov et al., 2016). Lastly, mitochondria are a significant source of ROS, which plays a role in redox signaling. ROS can directly modulate ion channel function, inducing changes in neuronal excitability, thus contributing to the intricate interplay between mitochondria and ion channels in regulating neuronal activity and excitability (Umare et al., 2021; V Frolov et al., 2016).

#### 6.2.1 Mitochondria and ion channels in epilepsy

**FIGURE 4:**
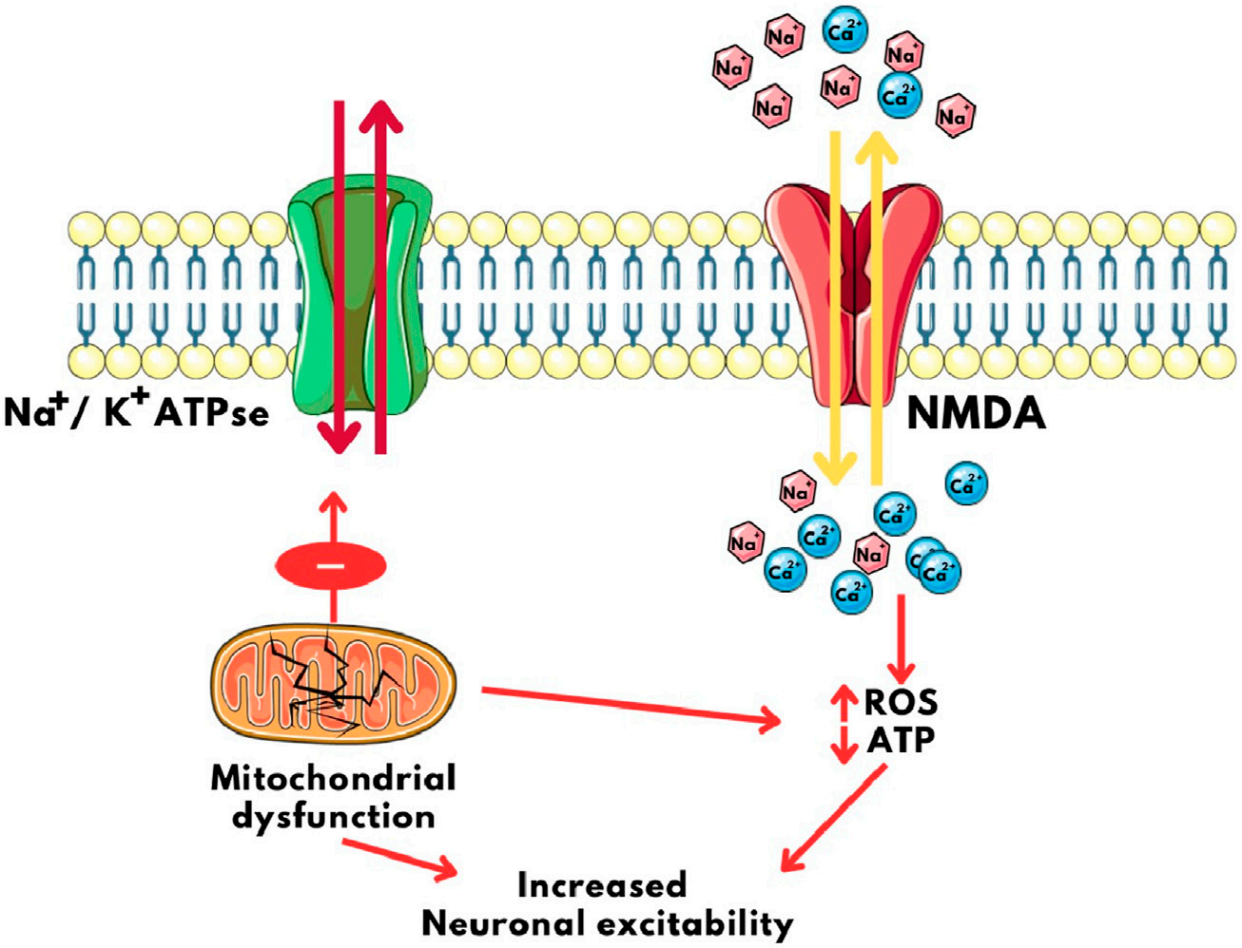
Calcium imbalance and Neuronal Excitability caused by mitochondrial disfunction. NMDA: N-Methyl D- Aspartate, ROS: Reactive Oxygen Species, ATP: Adenosine triphosphate, Na^+^/K^+^ ATPase: Sodium Potassium ATPase pump.

The interplay between mitochondria and ion channels becomes particularly significant in epilepsy. Mitochondrial dysfunction, a common feature of epilepsy, can disrupt the delicate balance of ion channel regulation, contributing to hyperexcitability and seizure generation. Mitochondrial dysfunction often results in reduced ATP production. This energy deficit compromises the function of the Na+/K+ pump, leading to membrane depolarization and increased neuronal excitability (Chen et al., 2022; Vezzani et al., 2011). Impaired mitochondrial calcium handling can disrupt calcium homeostasis in neurons. Elevated intracellular calcium levels can activate calcium-sensitive ion channels, exacerbating excitability and seizure susceptibility (Zündorf and Reiser, 2011; Gleichmann and Mattson, 2011). Mitochondrial dysfunction can lead to excessive ROS production. ROS can modulate ion channel activity, enhancing excitability and promoting seizure generation. For example, ROS can modulate NMDA receptor function, intensifying excitatory neurotransmission (Figure 4) (Umare et al., 2021; Tiwari et al., 2021)

#### 6.2.2 Mitochondrial calcium handling and excitability

Mitochondria serve a crucial role in controlling intracellular calcium concentrations. Utilizing the mitochondrial calcium uniporter (MCU) complex, mitochondria uptake and regulate calcium ions. This mechanism holds substantial implications for neuronal excitability. Mitochondria, as calcium sinks, efficiently buffer excessive cytoplasmic calcium, which is vital for preserving the function of ion channels and averting excitotoxicity (an occurrence where an excess influx of calcium causes neuronal harm) (Rharass et al., 2014; Li et al., 2020). Intra-mitochondrial calcium also influences the activity of enzymes involved in mitochondrial metabolism. This calcium-dependent regulation impacts ATP production and the generation of ROS, ultimately influencing neuronal excitability (Zorov et al., 2014). The dynamic modulation of excitability by mitochondrial calcium handling extends to various aspects of neuronal function. Mitochondria can influence neurotransmitter release by regulating calcium levels in presynaptic terminals, thereby shaping synaptic strength and overall neuronal network activity (Vos et al., 2010). Additionally, mitochondrial calcium uptake and release can impact the initiation and propagation of action potentials, contributing to changes in membrane potential and neuronal excitability. This multifaceted role of mitochondria in calcium handling underscores their significance in the complex regulation of neuronal excitability in health and disease, including epilepsy (Walters and Usachev, 2023).

#### 6.2.3 Role of mitochondrial calcium handling in epilepsy

Dysfunctional mitochondrial calcium handling stands out as a hallmark of epilepsy and significantly contributes to the pathophysiology of this neurological disorder. One critical consequence of impaired calcium regulation within mitochondria is excitotoxicity, a process where excessive calcium influx into neurons triggers cell damage and death (Verma et al., 2022; Vos et al., 2010; V Varga et al., 2015). This excitotoxicity not only exacerbates neuronal injury during seizures but also perpetuates a cycle of neural damage. Furthermore, altered mitochondrial calcium handling can lower the threshold for seizure initiation by disrupting membrane potential and synaptic transmission (Kann and Kovács, 2007; Panov et al., 2002; Pickrell et al., 2011). This disruption increases neuronal excitability, rendering the brain more susceptible to the spontaneous and synchronized firing of neurons characteristic of seizures. Additionally, the dysregulation of calcium levels within mitochondria can increase the production of ROS. These ROS can further perturb calcium handling and ion channel function, creating a positive feedback loop of hyperexcitability and oxidative stress, which plays a central role in the progression and perpetuation of epilepsy (Baev et al., 2022).

## 7 Mitochondrial epigenetics in epileptogenesis

Mitochondrial epigenetics in epileptogenesis explores how epigenetic modifications, such as DNA methylation, histone modifications, and non-coding RNA expression, influence mitochondrial function (Ren et al., 2023). These modifications can directly impact mitochondrial genes, leading to changes in energy metabolism, ROS generation, and mitochondrial biogenesis (Henshall and Kobow, 2015). Understanding the crosstalk between epigenetics and mitochondria sheds light on the molecular mechanisms underlying epileptogenesis, offering potential avenues for therapeutic interventions to restore mitochondrial function and mitigate neuronal hyperexcitability in epilepsy.

### 7.1 Epigenetic modifications and their influence on mitochondrial function

Epigenetic modifications play a critical role in regulating gene expression concerning mitochondrial function. These modifications, including DNA methylation, histone modifications, and non-coding RNAs, intricately shape the epigenetic environment, controlling the expression of genes associated with mitochondrial biogenesis, metabolism, and function. In epilepsy, disruptions in these epigenetic patterns are increasingly acknowledged as factors contributing to the abnormal neuronal excitability observed during seizures (Henshall and Kobow, 2015). DNA methylation, which entails adding methyl groups to cytosine residues in CpG dinucleotides, can directly impact mitochondrial function by regulating the expression of genes responsible for encoding mitochondrial proteins (Dostal and Churchill, 2019). For example, hypermethylation of the PGC-1α gene, a master regulator of mitochondrial biogenesis and function, has been linked to reduced mitochondrial density and compromised oxidative metabolism in epilepsy (Abu Shelbayeh et al., 2023). Histone modifications (acetylation, methylation, and phosphorylation) regulate chromatin structure and gene expression. In epilepsy, changes in histone acetylation and methylation patterns have been linked to alterations in gene expression, particularly those governing ion channel regulation and synaptic transmission. Significantly, these histone modifications can indirectly affect mitochondrial function by influencing the expression of nuclear-encoded mitochondrial genes, thereby impacting mitochondrial biogenesis and oxidative capacity (Boison and Rho, 2020). Non-coding RNAs, including microRNAs (miRNAs) and long non-coding RNAs (lncRNAs), have become vital regulators in epigenetic processes. In epilepsy, dysregulation of miRNAs directly affects mitochondrial genes, leading to compromised mitochondrial function, reduced ATP production, and increased oxidative stress levels. Moreover, lncRNAs have been demonstrated to influence mitochondrial dynamics and bioenergetics by interacting with nuclear-encoded mitochondrial genes, thereby introducing additional complexity to the epigenetic control of mitochondrial activity (Catanesi et al., 2020; Wang and Zhao, 2021). In summation, epigenetic modifications profoundly impact mitochondrial function through diverse mechanisms, revealing their central role in the molecular intricacies of epileptogenesis and offering potential avenues for therapeutic intervention aimed at restoring mitochondrial function and mitigating the hyperexcitability characteristic of epilepsy.

### 7.2 Epigenetics in epilepsy: Implications for mitochondrial involvement

The intricate interplay between epigenetics and epilepsy has illuminated a multifaceted landscape of molecular mechanisms driving epileptogenesis, with profound implications for mitochondrial involvement. Epilepsy, characterized by recurrent seizures and abnormal neuronal excitability, has increasingly been linked to epigenetic alterations, such as DNA methylation, histone modifications, and non-coding RNA expression. These epigenetic modifications exert far-reaching effects on gene expression patterns within neurons, influencing the expression of genes directly associated with mitochondrial function and homeostasis (Henshall and Kobow, 2015; Boison and Rho, 2020). In epilepsy, such epigenetic modifications can lead to mitochondrial dysfunction through various avenues, profoundly affecting neuronal excitability. One notable consequence is metabolic reprogramming, where altered epigenetic patterns steer neuronal energy metabolism away from oxidative phosphorylation and towards glycolysis. This metabolic shift diminishes mitochondrial ATP production, depriving neurons of the energy required for ion pump operation and overall excitability regulation. Moreover, epigenetically induced mitochondrial dysfunction contributes to increased production of ROS, exacerbating oxidative stress and further perturbing mitochondrial membrane integrity and electron transport chain function (Bhatti et al., 2017). This cascade of events significantly contributes to neuronal hyperexcitability, a hallmark of epilepsy.

Furthermore, epigenetic regulation extends to mitochondrial biogenesis, impacting mitochondrial density and function. Altered DNA methylation and histone modifications can either promote or hinder mitochondrial biogenesis, affecting the capacity for energy production and calcium buffering and thereby influencing neuronal excitability (Henshall and Kobow, 2015; Jiang et al., 2008; Van Vliet et al., 2007). Additionally, epigenetic changes can directly influence ion channel expression, disrupting ion homeostasis and membrane potential, ultimately promoting hyperexcitability and lowering the seizure threshold (Qureshi and Mehler, 2010). Collectively, these intricate interactions underscore the vital role of epigenetics in the pathophysiology of epilepsy and its direct implications for mitochondrial involvement. Understanding these molecular intricacies offers promising avenues for therapeutic intervention in epilepsy, with strategies aimed at restoring mitochondrial function and mitigating the aberrant neuronal excitability that characterizes this neurological disorder.

## 8 Therapeutic Strategies Targeting Mitochondria in Epilepsy

**FIGURE 5:**
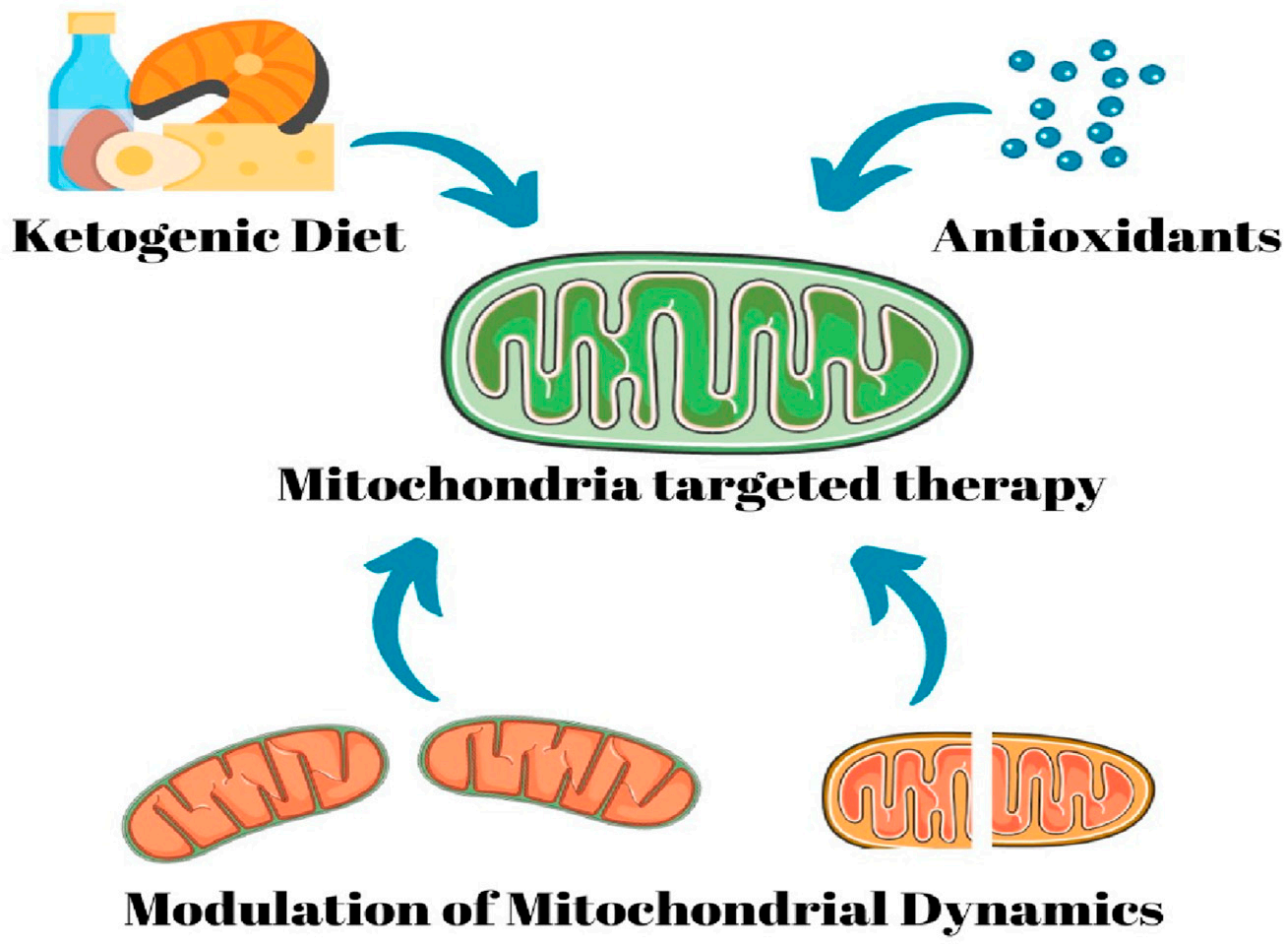
Therapeutic strategies targeting mitochondria in epilepsy.

Therapeutic strategies targeting mitochondria in epilepsy aim to restore mitochondrial function and mitigate neuronal hyperexcitability. These approaches include enhancing mitochondrial biogenesis, improving oxidative phosphorylation, and reducing oxidative stress. Modulating mitochondrial calcium handling and preserving membrane potential are also under investigation. Additionally, compounds like antioxidants and mitochondrial-targeted agents hold promise in attenuating mitochondrial dysfunction and its contribution to epileptogenesis (Catanesi et al., 2020; Madireddy and Madireddy, 2023). These emerging therapies represent a novel frontier in epilepsy treatment that potentially addresses the root causes of neuronal hyperexcitability and provides more effective mitochondria-focused interventions for individuals with epilepsy (Figure 5).

### 8.1 Mitochondria-targeted antioxidants

Mitochondria-targeted antioxidants represent a promising therapeutic approach for addressing mitochondrial dysfunction and oxidative stress in epilepsy. These compounds are designed to accumulate within the mitochondria, where they can neutralize ROS and protect mitochondrial components from oxidative damage. The rationale behind using mitochondria-targeted antioxidants in epilepsy is their potential to mitigate the harmful effects of excessive ROS production, a common feature of mitochondrial dysfunction in this neurological disorder (Umare et al., 2021). Mitochondria-targeted antioxidants typically contain an antioxidant molecule linked to a lipophilic cation, allowing them to accumulate selectively in the mitochondria due to the organelle’s negative membrane potential. Once inside the mitochondria, these antioxidants scavenge ROS, including superoxide and hydrogen peroxide, which are natural byproducts of oxidative phosphorylation (Yang et al., 2020; Mukhopadhyay et al., 2012). Firstly, they play a crucial role in reducing oxidative stress within the mitochondria, effectively curbing the accumulation of ROS. This reduction in oxidative stress is instrumental in mitigating mitochondrial dysfunction, thereby preserving the organelle’s pivotal functions in ATP production and calcium buffering. Secondly, these antioxidants contribute to neuroprotection by safeguarding mitochondrial integrity. In epilepsy, where seizures and oxidative stress often lead to neuronal damage and cell death, the preservation of mitochondria’s structural and functional integrity offers a promising strategy for shielding neurons from seizure-induced injury (Waldbaum and Patel, 2010a; Waldbaum and Patel, 2010b). Lastly, mitochondria-targeted antioxidants may indirectly influence neuronal excitability by maintaining ion channel function and cellular energy balance. This modulation of excitability, stemming from the preservation of mitochondrial function and reduction in oxidative stress, can potentially decrease the likelihood of seizure initiation and propagation (Apostolova and Victor, 2015). Collectively, these diverse benefits underscore the therapeutic potential of mitochondria-targeted antioxidants in managing epilepsy, addressing the symptoms and underlying cellular and molecular mechanisms contributing to this neurological disorder (Table 1).

**TABLE 1:** Mitochondria Targeted Antioxidant for the treatment of epilepsy.

Sr No.	Mitochondria-targeted antioxidant	Mechanism of action
1	MitoQ	It accumulates in mitochondria, scavenges ROS, and protects mitochondrial components from oxidative damage
2	SS-31	It targets mitochondria, reduces oxidative stress, and maintains mitochondrial membrane potential, preserving mitochondrial function
3	SkQR1	It accumulates in mitochondria, scavenges ROS, and protects mitochondrial DNA and proteins from oxidative damage
4	Szeto-Schiller (SS) Peptides	Selectively accumulate in mitochondria, reduce ROS production, and maintain mitochondrial membrane potential
5	MitoTEMPO	It accumulates in mitochondria, scavenges superoxide, and reduces oxidative stress, preserving mitochondrial function

### 8.2 Modulation of mitochondrial dynamics for seizure control

Modulation of mitochondrial dynamics represents an emerging strategy for seizure control in epilepsy (Cardoso et al., 2022). Therapeutic interventions aimed at restoring proper mitochondrial dynamics hold promise for mitigating seizure activity (Luo et al., 2020). Promoting mitochondrial fusion can enhance the organelle’s bioenergetic capacity and calcium buffering capabilities, potentially raising the seizure threshold (Uittenbogaard and Chiaramello, 2014). Conversely, encouraging mitochondrial fission may facilitate the removal of damaged mitochondria, reducing the generation of reactive oxygen species and oxidative stress, both associated with epileptogenesis (Chen et al., 2012). While the field of mitochondrial dynamics modulation for seizure control is still in its infancy, it offers an intriguing avenue for the development of innovative epilepsy therapies targeting the very core of mitochondrial dysfunction underlying this neurological condition.

### 8.3 Ketogenic diet and metabolic therapies

The ketogenic diet and related metabolic therapies have garnered substantial attention for their potential in managing epilepsy, particularly drug-resistant forms. The ketogenic diet is characterized by high-fat, low-carbohydrate, and moderate-protein intake, which induces a metabolic shift in the body, producing ketone bodies as an alternative energy source (Shaaban et al., 2023). Ketone bodies, such as beta-hydroxybutyrate, acetoacetate, and acetone, have been shown to exert neuroprotective effects, modulate neuronal excitability, and enhance mitochondrial function (Yang et al., 2019). These metabolic changes may help raise the seizure threshold and reduce seizure frequency in some individuals with epilepsy. Other metabolic therapies, including the modified Atkins diet and medium-chain triglyceride (MCT) oil supplementation, offer variations of the ketogenic approach, providing flexibility in dietary management (D’Andrea Meira et al., 2019; Borowicz-Reutt et al., 2024).While the mechanisms underlying the antiepileptic effects of these therapies are not fully understood, they likely involve a combination of factors, including increased mitochondrial efficiency, reduced oxidative stress, and altered neurotransmitter metabolism. Although the ketogenic diet and metabolic therapies may not be suitable for all epilepsy patients, they represent valuable adjunctive options, especially for those with drug-resistant epilepsy, offering a non-pharmacological approach to seizure control and improved quality of life.

## 9 Future directions and challenges

Advancements in mitochondrial research offer the potential for greater comprehension of the intricate connection between epilepsy and mitochondrial function. Future investigations may unveil novel mitochondrial targets for therapeutic intervention, refining treatment approaches for individuals affected by epilepsy. Pursuing more selective and effective mitochondria-targeted therapies and enhanced diagnostic tools to evaluate mitochondrial function in patients represents a critical area of study. Additionally, delving deeper into the role of mitochondrial genetics and epigenetics in epilepsy susceptibility could pave the way for personalized treatment strategies.

As mitochondrial-based therapies, such as mitochondrial transplantation and gene editing techniques like mitochondrial replacement therapy (MRT), progress, ethical considerations come to the forefront. Safety, long-term consequences, consent protocols, and equitable access to emerging treatments are vital concerns. Establishing ethical guidelines and robust regulatory frameworks is imperative to ensure the responsible advancement and integration of these therapies into clinical practice.

## 10 Conclusion

The intricate interplay between epilepsy and mitochondrial function highlights the complex nature of this neurological condition. Mitochondria play a central role in governing energy metabolism, calcium balance, and oxidative stress, which influence neuronal excitability and seizure susceptibility. With the prevalence of epilepsy in elderly populations growing, addressing this issue is increasingly vital. Despite significant strides in understanding these associations, challenges persist in translating these findings into effective treatments. The future of epilepsy management may hinge on personalized approaches targeting mitochondrial dysfunction. Advancements in research, innovative therapeutic strategies, and ethical considerations will be instrumental in advancing toward better outcomes and enhancing the quality of life for individuals grappling with epilepsy.
